# Joint tissue plasticity in hemophilia: insights from the Joint Activity and Damage Exam ultrasound protocol

**DOI:** 10.1016/j.rpth.2025.103290

**Published:** 2025-12-10

**Authors:** Parthiv Sheth, Khang Tong, Bilgimol Chumappumkal Joseph, Tina Manon-Jensen, Michael Glenzer, Isaac Nwi-Mozu, Peter Aguero, Bruno Steiner, Cindy Bailey, Doris V. Quon, Rebecca Kruse-Jarres, Euyhyun Lee, Morten Asser Karsdal, Lin Liu, Annette von Drygalski

**Affiliations:** 1 Division of Hematology/Oncology, Department of Medicine, University of California San Diego, La Jolla, California, USA; 2 Altman Clinical and Translational Research Institute, University of California San Diego, La Jolla, California, USA; 3 Nordic Biosciences, Herlev, Denmark; 4 Washington Center for Bleeding Disorders, Seattle, Washington, USA; 5 Orthopaedic Hemophilia Treatment Center, Orthopaedic Institute for Children Los Angeles, Los Angeles, California, USA; 6 Herbert Wertheim School of Public Health and Human Longevity Science, University of California San Diego, La Jolla, California, USA

**Keywords:** biomarkers, cartilage, hemophilia, hemarthrosis, ultrasonography

## Abstract

**Background:**

Progressive arthropathy is a debilitating condition that affects hemophilic joints as a consequence of hemarthroses. However, our understanding of the trajectory of potentially destructive processes at the tissue level remains limited and requires longitudinal imaging studies.

**Objectives:**

To longitudinally characterize ultrasonographic joint tissue changes in adults with hemophilia using the Joint Activity and Damage Exam (JADE) protocol, and to examine their relationships with joint health status, hemarthrosis, and serum biomarkers of tissue turnover.

**Methods:**

We prospectively studied adults with hemophilia (A or B) of any severity and associated arthropathy at 3 North American sites. We assessed joint health using the Hemophilia Joint Health Score (HJHS) and musculoskeletal ultrasound, as well as the Joint Activity and Damage Exam protocol for direct measurements of osteochondral alterations, cartilage, and soft tissue in the elbows, ankles, and knees at study entry and at ∼12 to 18 and ∼24 to 36 months thereafter. Reported hemarthrosis between study visits was verified by musculoskeletal ultrasound. We analyzed tissue rate changes in healthy (HJHS 0-3) and unhealthy (HJHS ≥ 4) joints, as well as in joints with and without hemarthrosis. Associations between tissue measurements and serially obtained serum biomarkers (C4M, PRO-C4, and C3M) were explored using logistic regression.

**Results:**

The median age (*N* = 44) was 36.5 years. Joints (133 healthy and 131 unhealthy at baseline) were evaluated serially. Only 16 joints experienced at least 1 hemarthrosis. At baseline, unhealthy joints had significantly thinner cartilage and increased osteochondral alterations and soft tissue swelling (all *P* values < .001). While individual joint tissues followed either a positive or negative trajectory of expansion or contraction over the ensuing 2 to 3 years, mean rate changes were not significantly different between healthy and unhealthy joints. Hemarthrosis resulted in a predilection for cartilage thinning and positive rate changes in PRO-C4 (*P* = .006).

**Conclusion:**

Ultrasonographic tissue measurements discriminated between healthy and unhealthy joints at baseline. Measurements revealed a dynamic pattern of tissue plasticity not only in healthy but also in unhealthy and bleeding joints, suggesting potential for reversibility of often-assumed irreversible intra-articular damage in hemophilic joints.

## Essentials

• The precise trajectory of tissue destruction and repair in hemophilic arthropathy is unknown.
• Longitudinal evaluation was performed with musculoskeletal ultrasound (JADE protocol).
• Tissue measurements demonstrated dynamic tissue plasticity, irrespective of joint health status.
• Tissue measurements revealed unanticipated repair of assumed irreversible cartilage damage.

## Visual Abstract

**Figure:**
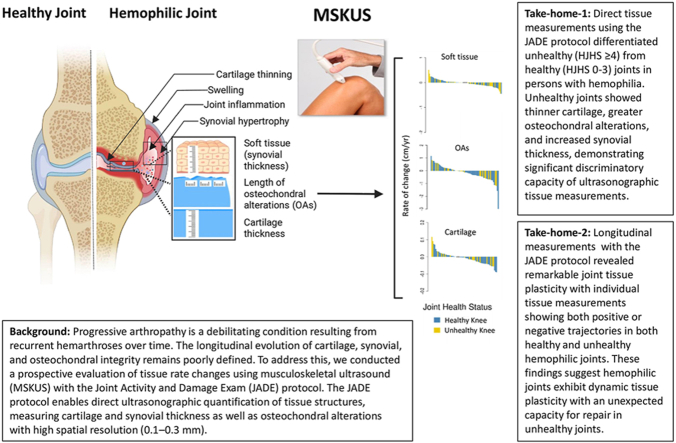

## 1 Introduction

Persons with hemophilia experience recurrent joint bleeding, affecting the elbows, ankles, and knees [1,2]. Recurrent intra-articular bleeding results in changes in the joint synovium, cartilage, and bone, which contribute to the development of hemophilic arthropathy (HA) [3]. Although HA is a common debilitating morbidity in persons with hemophilia, our understanding regarding the trajectory of this destructive process at the tissue level remains limited.

Traditionally, clinical scoring systems, such as the Hemophilia Joint Health Score (HJHS), which evaluate parameters including joint swelling, pain, and flexion/extension loss, have been used to identify and estimate the degree of arthropathy in persons with hemophilia [4,5]. However, the HJHS is based on clinical examinations and has a limited ability to discriminate among various tissue abnormalities. Therefore, ultrasonographic assessments are proposed as an alternative method for identifying the degree at the tissue level [6]. Musculoskeletal ultrasound (MSKUS) is sensitive to blood in the joint and is frequently used for rapid evaluation of hemarthrosis, but it can also be useful for tracking the progression of HA or joint changes longitudinally [[7], [8], [9], [10], [11]]. The Joint Activity and Damage Exam (JADE) is an MSKUS protocol that includes direct joint tissue measurements of cartilage thickness, osteochondral alterations, and soft tissue expansion at sentinel positions in the elbow, knee, and ankle. The JADE protocol has been validated previously in an iterative fashion following the Outcome Measures in Rheumatology guidelines [[12], [13], [14], [15], [16], [17]]. JADE measurements correlate well with clinical (HJHS) and functional (total arc) joint assessments consistently over time, with a high degree of intra- and interreader, as well as interoperator, reliability [[12], [13], [14]]. Sequential, cross-sectional assessments demonstrated that decreased cartilage thickness, increased length of osteochondral alterations, and greater soft tissue expansion were consistently associated with deteriorating HJHS and total arc in specific sentinel views, resulting in the JADE version 2 (v2) protocol [14].

However, joint tissue plasticity in both healthy and unhealthy hemophilic joints remains unknown. We thus used the JADE v2 protocol to prospectively quantify annual rate changes in joint tissue measurements in a cohort of adults with hemophilia. We also related ultrasonographic tissue measurement changes to clinical joint outcomes and to investigational serum biomarkers (PRO-C4, C4M, and C3M), which have previously been shown to be associated with HA disease activity in response to treatment [18]. Additionally, PRO-C4 and C4M previously demonstrated transient elevations during episodes of hemarthrosis in persons with hemophilia and in mouse models of hemophilia [16,19,20].

## 2 Methods

### 2.1 Patient selection

Adults with hemophilia (A or B) at 3 hemophilia treatment centers in the United States (Los Angeles Orthopaedic Institute for Children, University of California San Diego, and Washington Center for Bleeding Disorders) were recruited for the study. Inclusion criteria were age ≥18 years and at least 1 arthropathic joint, as evidenced by a Pettersson score ≥1 or a HJHS ≥ 4 [6,21].

There were no study exclusion criteria. Patients were followed prospectively for up to 36 months, with joint assessments and blood draws (for biomarker analysis) performed at baseline and during 2 subsequent study visits, each approximately 1 year apart. All selected patients signed written informed consent, and the study was approved by the ethics committees and/or institutional review boards of all 3 institutions.

### 2.2 Joint health evaluation

Joint health of both elbows, knees, and ankles was assessed by MSKUS using the JADE v2 protocol and the HJHS (version 2.1 [v2.1]) at 3 time points: study entry (baseline), at ∼12 to 18 months (midpoint), and at ∼24 to 36 months (final) [5,14]. A joint was deemed healthy if the baseline HJHS was 0 to 3 and unhealthy if the HJHS was ≥4 [21,22]. These evaluations were performed when patients were in their usual state of health. Subjects were also evaluated during acute painful joint episodes (within a 48-hour window) with confirmation of hemarthrosis by MSKUS.

We used the HJHS v2.1 (HJHS per joint: 0 best, 20 worst; total HJHS for 6 joints combined: 0 best, 120 worst) [5]. The HJHS v2.1 is an established outcome measure that provides a clinical score for each joint, summarizing swelling, duration of swelling, pain, strength, loss of range of motion, muscle atrophy, and crepitus. HJHSs were performed by a licensed physical therapist with >5 years of general practice experience and at least 2 years of experience with persons with hemophilia (B.S., P.A., and C.B.). The physical therapists were trained in HJHS acquisition according to instructions and guidance provided in online training and video modules developed by the International Prophylaxis Study Group [23].

### 2.3 MSKUS imaging

MSKUS measurements were performed using a GE Logiq S8 high-resolution ultrasound machine (General Electric) and an 8 to 15 MHz high-frequency linear transducer. At each participating institution, images were obtained by hemophilia providers with at least 3 years of experience and who had been formally trained in MSKUS through the University of California San Diego Continuing Medical Edcuation accredited course [24]. Two providers (A.v.D. and B.S.) are certified in MSKUS by the Alliance for Physician Certification & Advancement, and P.A. and C.B. were in the process of certification (P.A. is now certified). Images delineated in JADE v2 were utilized for soft tissue, osteochondral, and cartilage measurements [14]. Measurements were performed by a single provider at each institution (P.A., B.S., and C.B.) after study completion. MSKUS evaluation during painful episodes included sonopalpation to evaluate compressibility and displacement of intra-articular material within the joint recesses, distinguishing simple from complex (bloody) effusions [7,8].

### 2.4 Biomarker analysis

Blood was drawn by venipuncture into sodium citrate-coated tubes and processed per standard protocol. Plasma samples were aliquoted and stored at −80 °C until analyzed. All biomarkers (Supplementary Table S1) were measured using a validated competitive enzyme-linked immunosorbent assay manufactured by Nordic Biosciences, which uses monoclonal antibodies targeting specific neo-epitope antigens of various collagens, as described previously [16,25,26]. A calibration curve was plotted using a 4-parameter logistic curve fit [16].

### 2.5 Statistical analysis

Descriptive statistics were reported as medians with interquartile ranges (Q1-Q3) for continuous variables and counts (%) for categorical variables. Baseline comparisons of tissue measurements between healthy and unhealthy joints were conducted using a linear mixed-effects model with a random intercept to account for correlations between joints (left- and right-sided) from the same subject.

Annual rates of change were calculated by subtracting final tissue measurements from baseline measurements and then dividing this value by the number of years the individual was followed in the study. Waterfall plots were then generated for each tissue to visualize the annual rate of change for each joint.

To examine differences in longitudinal change in tissue measurements between healthy and unhealthy joints, linear mixed-effects models with random intercepts were utilized to account for the correlation arising from repeated measures across the study periods and from each participant having left- and right-sided joints. Time, joint health status, and an interaction between time and joint health status were included as fixed effects. Annualized rates of change and 95% CIs, stratified by joint health status, were estimated from the fitted mixed-effect models. Baseline demographic variables, including age, sex, race, body mass index (BMI), hemophilia type/severity, treatment, and hemophilia treatment center, were assessed as potential covariates in the model. From a full model including all demographic variables with *P* < .20 in univariable screening, we utilized backward elimination, iteratively removing insignificant baseline demographic variables until all variables had *P* < .10. Joints with no measurable cartilage thickness were considered to have a value of 0 in the analysis. Sensitivity analysis was conducted by removing joints.

Correlations of annual rate changes between tissue types of the same joint type were calculated using repeated-measures correlation [27,28]. The relationship between the combined annual rate changes across all tissue types and whether a biomarker experienced a positive change was examined using a logistic regression model. Combined annual rate changes were calculated by averaging left and right joint measurements and summing across the elbow, ankle, and knee.

Statistical analyses were conducted using SAS v9.4 (SAS Institute Inc) and R v4.3.1 (R Core Team), with a 2-sided *P* < .05 indicating significance.

## 3 Results

### 3.1 Patient and joint characteristics

Baseline patient characteristics are summarized in Table 1. Among the 44 enrolled study participants, the median age was 36.5 (Q1-Q3, 28.8-49.0), and the median BMI was 26 kg/m^2^ (Q1-Q3, 23.2-29.6). Most patients had severe hemophilia (81.8%) and were managed with regular factor (F)VIII prophylaxis (86.4%). Of note, 2 patients were receiving emicizumab prophylaxis, and 2 patients had undergone experimental FVIII gene therapy without exogenous factor needs.

**Table 1:** Baseline patient characteristics.

Characteristic	Total patients (*N* = 44)
**Age (y)**	36.5 (28.8-49.0)
**BMI (kg/m^2^)**	26.0 (23.2-29.6)
**Sex**	
Male	44 (100)
Female	0 (0)
**Race/ethnicity**	
White	26 (59.1)
Hispanic	7 (15.9)
Black	4 (9.1)
Asian	2 (4.5)
Other	5 (11.4)
**Hemophilia treatment center**	
Los Angeles	14 (31.8)
Seattle	15 (34.1)
San Diego	15 (34.1)
**Hemophilia type**	
A	35 (79.5)
B	9 (20.5)
**Severity**	
Mild	1 (2.3)
Moderate	7 (15.9)
Severe	36 (81.8)
**Treatment**	
Factor prophylaxis	38 (86.4)
Factor episodic only	2 (4.5)
Emicizumab prophylaxis	2 (4.5)
Gene therapy	2 (4.5)

Values are expressed as median (quartile 1-quartile 3) for continuous variables and as *n* (%) for categorical variables.

BMI, body mass index.

At baseline, 133 joints were deemed healthy, and 131 were unhealthy. Supplementary Tables S2 and S3 summarize joint-specific and total HJHS findings. The HJHSs for healthy ankles, knees, and elbows worsened over time from baseline to study exit; however, statistical significance was reached only for ankles and knees (both *P* values < .01). Of note, the median scores all remained within the healthy range. There was no significant HJHS worsening noted for unhealthy joints. The total median baseline HJHS (excluding the global gait score) was 24 (Q1-Q3, 16-38), and the median final HJHS was 26 (Q1-Q3, 15-35; *P* = .66).

### 3.2 Baseline tissue measurements

Tissue measurements at baseline are shown in Table 2. Compared with healthy joints at baseline, unhealthy joints demonstrated thinner median cartilage (elbow: 0.09 cm vs 0.02 cm; ankle: 0.07 cm vs 0 cm; knee: 0.29 cm vs 0.22 cm), longer median osteochondral alterations (elbow: 3.00 cm vs 4.41 cm; ankle: 1.95 cm vs 2.94 cm; knee: 3.05 cm vs 3.70 cm), and greater median soft tissue proliferation (elbow [fat pad thickness]: 1.81 cm vs 2.63 cm; ankle [capsular thickness]: 0.47 vs 0.59 cm; knee [lateral synovial thickness]: 0.18 cm vs 0.30 cm; knee [medial synovial thickness]: 0.33 cm vs 0.47 cm). The differences among these tissue measurements were all statistically significant (all *P* values < .001), indicating that these measurements enabled differentiation between joints deemed healthy or unhealthy by clinical HJHS.

**Table 2:** Baseline tissue measurements among healthy joints (Hemophilia Joint Health Score 0-3) and unhealthy joints (Hemophilia Joint Health Score ≥ 4)

Joint	Tissue	*N*	Healthy (cm) median (Q1-Q3)	*N*	Unhealthy (cm) median (Q1-Q3)	*P* value^a^
**Elbow**	**Cartilage thickness**	51	0.09 (0.08-0.12)	32	0.02 (0.00-0.05)	<.001
	**Osteochondral alterations**	51	3.00 (1.98-3.84)	32	4.41 (3.68-4.83)	<.001
	**Fat pad thickness**	51	1.81 (1.38-2.05)	35	2.63 (2.34-3.24)	<.001
**Ankle**	**Cartilage thickness**	30	0.07 (0.05-0.09)	53	0.00 (0.00-0.04)	<.001
	**Osteochondral alterations**	30	1.95 (1.40-2.35)	58	2.94 (2.46-3.35)	<.001
	**Capsular thickness**	30	0.47 (0.32-0.60)	58	0.59 (0.44-0.91)	<.001
**Knee**	**Cartilage thickness**	49	0.29 (0.24-0.34)	22	0.22 (0.08-0.27)	<.001
	**Osteochondral alterations**	49	3.05 (2.50- 3.49)	23	3.70 (3.08-4.62)	<.001
	**Lateral synovial thickness**	50	0.18 (0.13-0.24)	38	0.30 (0.25-0.44)	<.001
	**Medial synovial thickness**	50	0.33 (0.25-0.43)	38	0.47 (0.39-0.66)	<.001

N, number of joints; Q, quartile.

a *P* values for comparisons of baseline tissue measurements between healthy and unhealthy joints are based on linear mixed-effects models.

### 3.3 Annualized rate changes of tissue measurements

**Figure 1:**
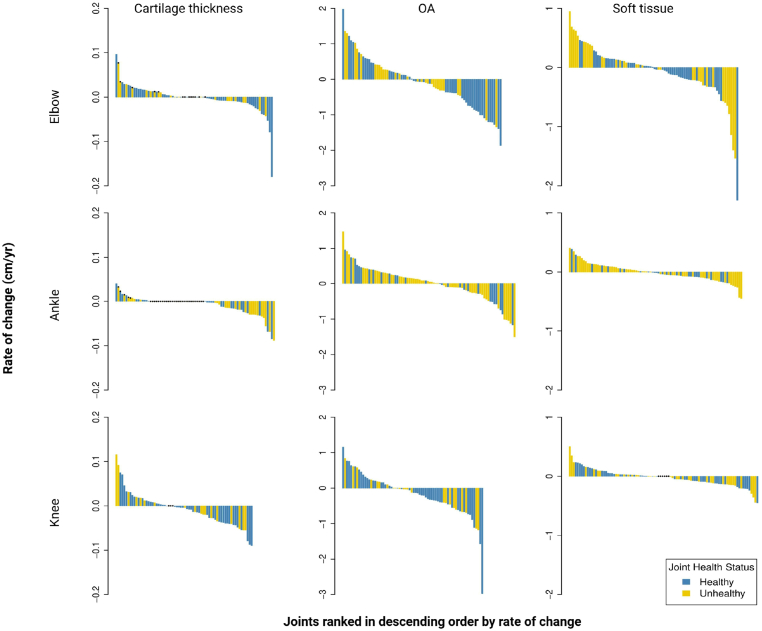
Waterfall plots for each tissue measurement ranked in descending order by rate of change, stratified by joint health status. Dots indicate joints with no measurable cartilage thickness at baseline. OA, asteochondral alteration.

The calculated rate change for each individual joint for measured osteochondral alterations, soft tissue, and cartilage thickness was plotted in descending order and stratified by baseline joint health status (Figure 1). Irrespective of joint type (elbow, knee, or ankle) and/or joint health status, we observed both positive and negative rate changes. The mean rate changes were nonsignificant and nondirectional, with the exception of cartilage measurements in unhealthy ankles (−0.008 cm/y; 95% CI, −0.013, −0.003; Table 3). Overall, there was no statistically significant difference in rate changes between healthy and unhealthy joints in all tissues. While individual joint tissues followed either a positive or negative rate trajectory, there were no significant correlations between the rate changes of cartilage, osteochondral, or soft tissue measurements within the same elbow, ankle, or knee. For example, in the elbow, the rate change in osteochondral alternations lacked associations with rate changes in cartilage or soft tissue thickness (repeated measures Pearson rho = −.18 and –.03, respectively). However, weak, nonsignificant associations were observed between ankle osteochondral alterations and capsular thickness (rho = .22; 95% CI, −.09, .48), and between knee cartilage thickness and combined synovial thickness (medial and lateral recesses; rho = −.27; 95% CI, −.57, .10).

**Table 3:** Annualized rates of change among healthy joints (Hemophilia Joint Health Score 0-3) and unhealthy joints (Hemophilia Joint Health Score ≥ 4).

Joint	Tissue	Joint health status	*N*	Annual rate of change (cm)	95% LCLM (cm)	95% UCLM (cm)	*P* value^a^
**Elbow**	**Cartilage thickness**	**Healthy**	51	−0.004	−0.009	0.001	.28
		**Unhealthy**	32	0.000	−0.006	0.006	
**Elbow**	**Osteochondral alterations**	**Healthy**	51	−0.139	−0.282	0.004	.16
		**Unhealthy**	32	0.030	−0.159	0.216	
**Elbow**	**Fat padthickness**	**Healthy**	51	−0.068	−0.159	0.023	.74
		**Unhealthy**	35	−0.092	−0.205	0.020	
**Ankle**	**Cartilage thickness**	**Healthy**	30	−0.004	−0.010	0.001	.35
		**Unhealthy**	53	−0.008	−0.013	−0.003	
**Ankle**	**Osteochondral alterations**	**Healthy**	30	0.067	−0.058	0.192	.78
		**Unhealthy**	58	0.044	−0.062	0.150	
**Ankle**	**Capsular thickness**	**Healthy**	30	−0.029	−0.074	0.016	.42
		**Unhealthy**	58	−0.005	−0.041	0.032	
**Knee**	**Cartilage thickness**	**Healthy**	49	−0.004	−0.014	0.005	.69
		**Unhealthy**	22	−0.001	−0.015	0.013	
**Knee**	**Osteochondral alterations**	**Healthy**	49	−0.134	−0.282	0.014	.92
		**Unhealthy**	23	−0.119	−0.340	0.101	
**Knee**	**Lateral synovial thickness**	**Healthy**	50	−0.009	−0.034	0.017	.21
		**Unhealthy**	38	0.015	−0.012	0.042	
**Knee**	**Medial synovial thickness**	**Healthy**	50	−0.010	−0.041	0.020	.78
		**Unhealthy**	38	−0.017	−0.049	0.016	

95% LCLM, 95% lower confidence limit; 95% UCLM, 95% upper confidence limit; N, number of joints.

a Annual rate of change, 95% confidence limits, and *P* values for comparison of the rate of change between healthy and unhealthy joints are based on linear mixed-effects models.

To further determine whether baseline variables (age, sex, race, BMI, hemophilia type/severity, prophylaxis management, and hemophilia treatment center) influenced tissue measurement rate changes, multivariable linear mixed-effects regression was applied. The analysis did not reveal any confounding associations between these variables and rate changes in tissue measurements.

### 3.4 Joints with hemarthrosis

**Figure 2:**
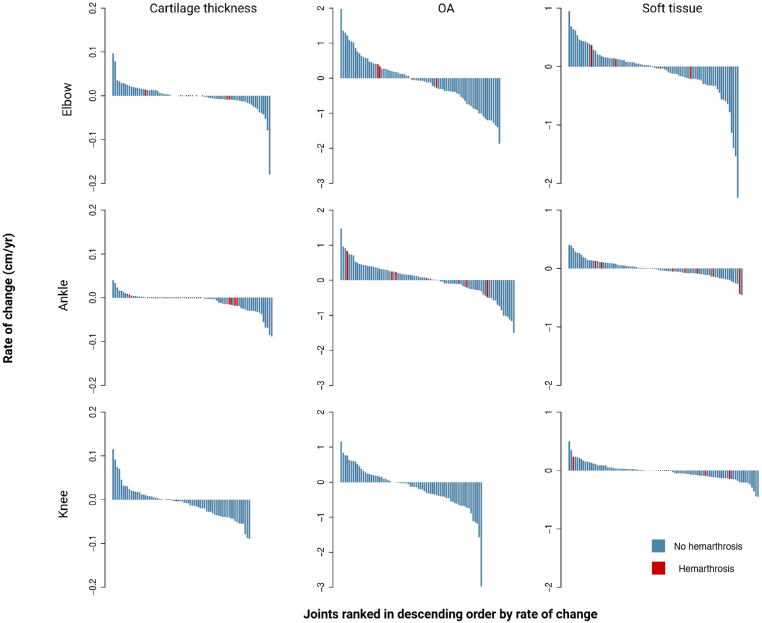
Waterfall plots for each tissue measurement ranked in descending order by rate of change, stratified by joints with and without hemarthrosis. Dots indicate joints with no measurable cartilage thickness at baseline. OA, asteochondral alteration.

Of the 264 joints evaluated longitudinally, 248 (94%) experienced no acute hemarthrosis (zero bleeds), indicating a cohort well controlled on prophylaxis. However, 16 joints (4 elbows, 8 ankles, and 4 knees) experienced at least 1 acute hemarthrosis confirmed by MSKUS. Notably, the HJHSs for these elbows and knees worsened, while the ankles remained stable throughout the study period (Supplementary Table S4). Rate changes for osteochondral alterations and soft tissue expansion were comparable to those observed in joints without hemarthrosis (Figure 2). However, cartilage rate changes in joints affected by hemarthrosis appeared to have a predilection for thinning (Figure 2). Specifically, among 13 joints with hemarthrosis and measurable cartilage at baseline (cartilage absent in 3 joints), 7 (2 elbows, 4 ankles, and 1 knee) demonstrated cartilage thinning.

### 3.5 Synovial basement membrane and interstitial turnover markers

**Figure 3:**
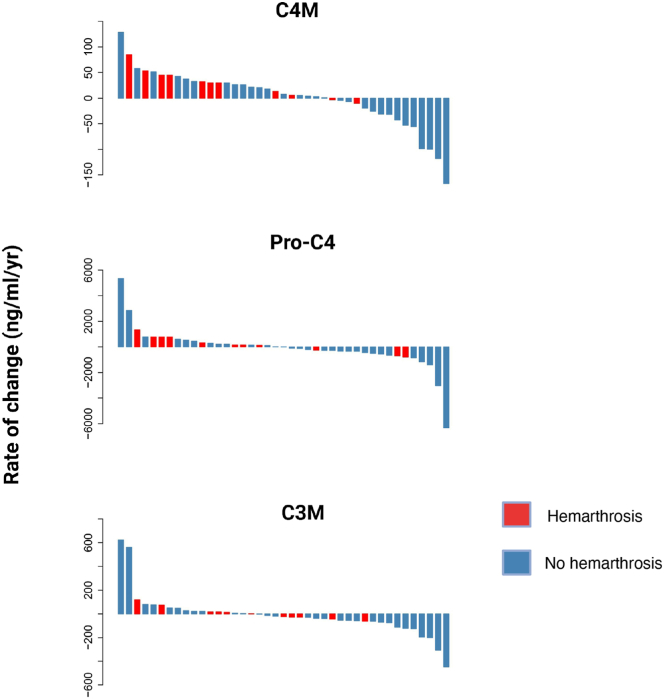
Waterfall plots for measured biomarkers ranked in descending order by rate of change.

Longitudinal analysis of biomarkers was possible in 41/44 persons with hemophilia with available samples (30 without hemarthrosis; 11 with at least 1 hemarthrosis). Annualized rate changes in persons with hemophilia, with and without hemarthrosis, are shown in Table 4. Notable rate changes were evident for the basement membrane turnover markers C4M and PRO-C4. Among the 11 persons with hemophilia and hemarthrosis, rate changes for C4M and PRO-C4 were positive in 9 (82%) and 8 (73%), respectively (Figure 3). Among the 30 persons with hemophilia without hemarthrosis, positive rate changes for C4M and PRO-C4 were noted in only 17 (57%) and 11 (37%) persons with hemophilia, respectively. For PRO-C4, the extent of rate change was significantly more positive in persons with hemophilia experiencing hemarthrosis (381 ng/mL/y; 95% CI, 65.1, 698) than in those without hemarthrosis (−179 ng/mL/y; 95% CI, −392, 35.6; *P* value for the difference in rates = .006). Conversely, C4M had a more modest positive rate of change in persons with hemophilia experiencing hemarthrosis (6.33 ng/mL/y; 95% CI, −11.8, 24.4), which did not differ significantly from that in persons without hemarthrosis (4.4 ng/mL/y; 95% CI, −8.20, 17.0). The interstitial turnover marker, C3M, had positive and negative rate changes with a relatively equal distribution, with no significant difference between persons with hemophilia with and without hemarthrosis (*P* = .07). Therefore, it appeared that PRO-C4 was most affected by hemarthrosis, while C4M and C3M were not.

**Table 4:** Annualized changes in biomarker rates among persons with hemophilia with and without hemarthrosis.

Biomarker	Hemarthrosis	*N*	Annual rate of change (ng/mL/y)	95% LCLM (ng/mL/y)	95% UCLM (ng/mL/y)	*P* value^a^
**C4M**	No	30	4.42	–8.20	17.0	.87
	Yes	11	6.33	–11.8	24.4	
**PRO-C4**	No	30	–179	–393	35.6	.006
	Yes	11	381	65.1	698	
**C3M**	No	30	–18.3	–41.5	4.87	.07
	Yes	11	21.4	–12.8	55.7	

95% LCLM, 95% lower confidence limit; 95% UCLM, 95% upper confidence limit; N, number of people with hemophilia.

a Annual rate of change, 95% confidence limits, and *P* values for comparison of the rate of change in persons with hemophilia with at least 1 hemarthrosis. None are based on linear mixed-effects models.

**Figure 4:**
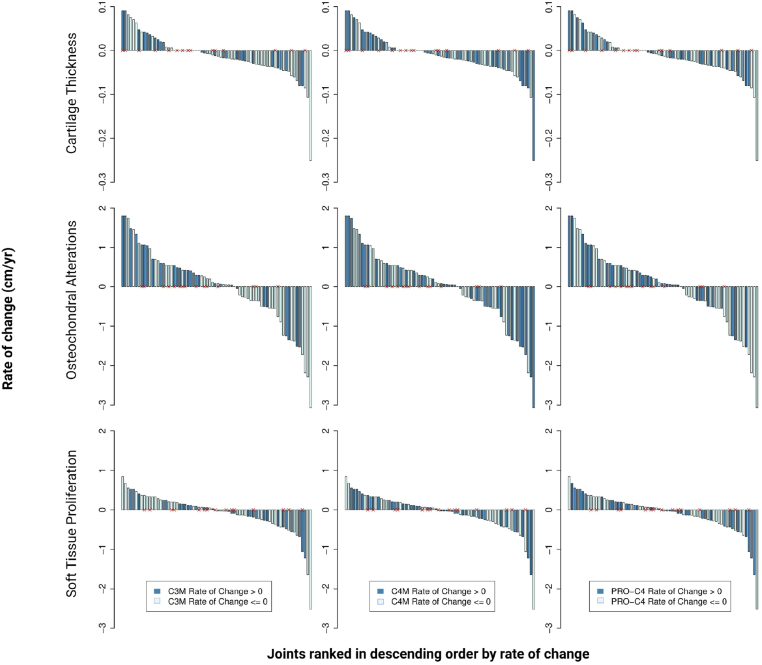
Waterfall plots demonstrating composite joint measurement rate changes for all joints (elbows, ankles, and knees), irrespective of baseline joint health status (y-axis). Patients are ranked in descending order by rate of change (x-axis), stratified by positive or negative biomarker rate of change (C3M, C4M, and pro-C4). Red Xs denote persons with hemophilia who experienced at least 1 joint hemarthrosis.

We then examined associations between rate changes in soft tissue measurements and biomarker values. The composite rate change in cartilage thickness, soft tissue thickness, or osteochondral alterations across all joints was compared between persons with hemophilia with and without positive rate changes in biomarker levels. There were no significant associations between rate changes in composite cartilage, osteochondral, or soft tissue measurements and rate changes in C4M, PRO-C4, or C3M, irrespective of hemarthrosis during the study period (Figure 4).

## 4 Discussion

This study provides the first sonographic evidence that hemophilic joints undergo dynamic intra-articular tissue changes with unanticipated plasticity, irrespective of joint health status. These observations were enabled by MSKUS tissue measurements using the JADE protocol v2 and advanced high-resolution equipment, which has been well established to achieve a minimum structural resolution of 0.1 to 0.3 mm with an 8 to 15 MHz transducer [14,[29], [30], [31]]. Measurement validity was demonstrated, as measurements were performed by well-trained providers who have previously shown to have high interoperator and interreader reliability, as well as serial measurement consistency over time within the same cohort [[12], [13], [14]]. Further, the validity and reliability of the measurements in this study were supported by their discriminatory capacity to discern between healthy and unhealthy joints at baseline, revealing significant differences in cartilage thickness, osteochondral alterations, and soft tissue expansion. Unhealthy joints had thinner cartilage, longer osteochondral alterations, and greater soft tissue expansion than healthy joints. The term “soft tissue expansion” rather than “synovial proliferation” is preferred since MSKUS has limited sensitivity for soft tissue discrimination in advanced HA [15]. Since the annualized rate-of-change estimates generated by the mixed-effects models may fall below the known spatial resolution of high-frequency ultrasound, these small values should be interpreted as indicators of trajectory and directionality rather than exact quantitative differences.

Several observations here advance our understanding of intra-articular tissue changes in HA. First, it was notable that reparative tissue changes can occur in unhealthy joints. Measurement rate changes may be expected from soft tissue expansion or regression, as soft tissue (eg, synovium) is biologically active [32,33]. However, a 2- to 3-year trajectory toward a decrease in osteochondral alterations or an increase in cartilage thickness in approximately half of the unhealthy joints was unexpected. This observation suggests a potential for chondral regenerative capacity to fill defects at the osteochondral surface and to improve cartilage thickness. While these changes were subtle (∼1/100th to 1/10th mm for cartilage; ∼1/10th to 1 mm for osteochondral alterations), they occurred at a similar rate to those in healthy joints, indicating that unhealthy hemophilic joint tissues can maintain plasticity and a capacity for chondral repair. Second, it was notable that tissues in healthy joints demonstrated bidirectional changes in tissue measurements, indicating dynamic fluctuations, even in adults with a mature skeleton and completed ossification of cartilaginous tissue. This observation may be important since surprisingly little is known about local cartilage maintenance in relation to daily “wear and tear” (in contrast to cartilage injury repair), with recent cellular and molecular studies implicating friction-related synovial detachment and attachment to cartilage defects, followed by chondrogenic differentiation [34,35].

These findings provide proof of principle that unhealthy cartilage in hemophilic joints may be more adept at repair than commonly assumed. For instance, given a healthy cartilage thickness of ∼2 to 3 mm in the adult knee, an annual thickness change rate of ∼0.1 mm would result in ∼0.3 mm of loss or gain over a 2- to 3-year period [36]. Although calculated cartilage rate changes fell below the established 0.1 to 0.3 mm resolution of modern ultrasound, the median individual cartilage measurements were approximately 1.2 mm (excluding joints with no measurable cartilage) and correlated well with the HJHS, as previously discussed [13,14]. Nonetheless, the exact threshold for the biological significance of these rate changes remains uncertain and may depend not only on cartilage thickness but also on cartilage quality, with relative depletion of matrix molecules such as glycosaminoglycans [34,37]. Eventually, such trajectories may bear clinical relevance and inform management decisions; however, it must be kept in mind that normal wear and tear accounts for ∼3% to 5% of annual cartilage volume in adults [34]. Of note, there was no directional correlation between rate changes across different tissues, demonstrating that soft tissue contraction or expansion, osteochondral alterations, and cartilage thickness changes were not linked unidirectionally (for instance, soft tissue swelling, increasing osteochondral alterations, and decreasing cartilage thickness; all findings indicating deteriorating joint health status). Instead, tissue changes occurred in a bidirectional and dyssynchronous fashion, suggesting a dynamic intra-articular environment without strict interdependence of tissue alterations (at least in the short term).

We previously demonstrated that the HJHS and functional arc correlated with absolute tissue measurements at fixed intervals at each study time point, highlighting the biological relevance of tissue measurements [14]. However, it was not possible to determine which of the intra-articular tissue changes (or combination thereof) may drive HJHS deterioration, as HJHSs were relatively unaffected over the study period. HJHSs remained stable in unhealthy joints, and while they deteriorated in healthy knees and ankles, they did not cross from healthy (score 0-3) to unhealthy (score ≥ 4). The HJHS is a semiquantitative estimation of hemophilic joint health that may also be influenced by extra-articular structures, such as tendons and muscles, as well as other personal health factors. Although the HJHS is the most widely used clinical joint health score, it has been deemed insensitive to joint health changes, prompting efforts to develop more sensitive tools [38]. Therefore, scoring systems such as the HJHS may not align with subclinical ultrasonographic tissue rate changes. Hence, the overall clinical context will be paramount in determining whether a tissue change trajectory, irrespective of HJHS change, should inform management decisions. Similarly, it remains unknown how such tissue changes may affect future joint function, which is frequently assessed using the Functional Independence Score in Hemophilia.

Our study cohort comprised adults with hemophilia with a wide spectrum of hemophilic joint disease (implied by a median HJHS of 24), consistent with suboptimal bleed control in childhood due to limited access to prophylaxis. In the United States, prophylaxis was not the standard of care prior to ∼2010, until its benefits were proven by Manco-Johnson et al.’s [39] landmark study. Of note, relatively few bleeding events occurred during the study period, indicating a well-controlled cohort on diverse prophylaxis regimens and relatively stable HJHSs. Dynamic tissue rate changes, related to reparative or destructive processes, may therefore differ in patients or joints with frequent bleeding episodes or with episodic treatment triggered by hemarthrosis. To shed light on tissue dynamics in joints with hemarthrosis, we analyzed tissue rate changes in joints experiencing hemarthrosis and compared them with those without hemarthrosis. A similar distribution of osteochondral and soft tissue rate changes was observed in joints with and without hemarthrosis; however, joints with hemarthrosis appeared to show a slight predisposition toward cartilage thinning, as did cartilage in unhealthy ankles. The low frequency of hemarthroses limited further analysis. However, it appears that occasional joint bleeds did not significantly affect the overall trajectory of tissue changes or joint health. This observation aligns well with the notion of tissue plasticity, likely resulting in tissue repair.

Furthermore, biomarker analysis demonstrated accelerated PRO-C4 turnover in persons with hemophilia who experienced hemarthrosis. This is consistent with previous observations in the acute bleed setting and in persons with hemophilia with suboptimal bleed control [16,18]. PRO-C4 is a turnover product of type IV basement membrane collagen, thought to originate from synovial vascular remodeling caused by joint bleeding and synovial inflammation [16,18]. This biomarker has previously been shown to be responsive to bleeding control in the acute bleeding setting in humans and mice [16,[18], [19], [20]]. Notably, alterations in type IV collagen metabolism are linked to disease activity, radiographic progression, and therapeutic response to tocilizumab in patients with rheumatoid arthritis, a joint disease that, like HA, involves chronic synovial inflammation and progressive joint damage [40]. The continuous biomarker turnover in the patients affected by hemarthrosis is therefore interesting and infers additional subclinical bleeding, underreported additional painful episodes, and/or ongoing vascular remodeling in the context of synovitis. While these biomarker findings remain exploratory, PRO-C4 seems to emerge as an indicator of ongoing synovial vascular tissue turnover and repair, with potential for clinical development and standardization as therapeutic response elements [41].

This study has several limitations, including the lack of information on the smallest detectable change by ultrasound and its clinical significance. Other limitations are mostly inherent to cohort heterogeneity. Patients exhibited a wide range of joint morbidity, used different prophylactic regimens (ranging from various clotting factor products and individualized infusion schedules to nonfactor products and gene therapy), and were followed at 3 different centers. The number of patients (*N* = 44) was relatively small. Although it approached cohort sizes comparable to those in drug trials for hemophilia, as a rare disease, it was not suitable for determining product effects. On the other hand, it may be argued that the type of prophylaxis is irrelevant to proof-of-principle observations and that the study is strengthened by its real-world nature and prospective multicenter approach, with no measurable center effect. Due to the relative lack of frequent bleeding events (a desirable outcome of any prophylaxis), dynamic tissue rate changes in joints affected by frequent or consecutive hemarthroses remain unexplored. Additionally, the degree to which subacute or nonsymptomatic bleeding may have influenced measurements is unknown. Since only reported painful joint events were examined by MSKUS to verify bleeding, unreported events may have been missed. However, we believe this number is low, based on our clinical practice patterns with patients who are well informed regarding the importance of reporting joint pain to optimize routine management. Of note, our cohort included only adult patients, and the reproducibility of these results in pediatric patients has yet to be determined.

## 5 Conclusions

Our observations provide proof of concept that hemophilic joints exhibit dynamic tissue plasticity throughout adulthood, demonstrating potential for osteochondral and cartilage repair in unhealthy joints. Serial sonographic intra-articular tissue measurements using the JADE v2 protocol, validated for hemophilic joint assessments, may allow for the detection of subclinical trajectories of soft tissue, osteochondral, and/or cartilage changes, and biomarkers, such as PRO-C4, may identify suboptimal bleed control. Together, these observations are hypothesis-generating and could provide innovative tools to inform clinical decision-making and/or serve as outcome measures in clinical studies.
