# Supplementary material for: Joint tissue plasticity in hemophilia: insights from the Joint Activity and Damage Exam ultrasound protocol
Source: Res Pract Thromb Haemost. 2025 Dec 10;10(1):103290. doi: 10.1016/j.rpth.2025.103290 (PMC12805341; doi:10.1016/j.rpth.2025.103290)
Supplement: Supplementary Tables [file mmc1.docx]

**Supplementary Table S1.** Description of serum biomarkers used in this study

| Biomarker | Specification | Function | Tissue of origin |
| --- | --- | --- | --- |
| C3M | A fragment of type III collagen released by MMP. Fibrillar collagen measuring soft tissue inflammation and destruction | ECM degradation,  Tissue inflammation | Interstitial |
| C4M | A fragment of type IV collagen released by MMP. Network forming collagen measuring basement membrane destruction | ECM degradation, Basement membrane degradation | Basement membrane |
| PRO-C4 | A fragment of the internal 7S domain of type IV collagen. Network forming collagen measuring epithelial cell-mediated basement membrane formation | ECM formation, Basement membrane synthesis | Basement membrane |

Abbreviations: C3M, type III collagen degradation marker; C4M, type IV collagen breakdown marker; PRO-C4, procollagen IV

**Supplementary Table S2.** Hemophilia Joint Health Score (HJHS) Characteristics by Joint

| Joint  (N = 264) | Healthy [HJHS 0-3] | | | | Unhealthy [HJHS ≥4] | | | |
| --- | --- | --- | --- | --- | --- | --- | --- | --- |
|  | **N** | **Baseline HJHS** | **Final HJHS** | **P-value^b^** | **N** | **Baseline HJHS** | **Final HJHS** | **P-value^b^** |
| Elbow | 53 | 0 [0-1]^a^ | 0 [0-1] | 0.08 | 35 | 8 [ 7-10] | 7 [6-10] | 0.20 |
| Ankle | 30 | 1 [0-1] | 2 [1-6] | <0.001 | 58 | 7 [6-10] | 8 [5-10] | 0.35 |
| Knee | 50 | 0 [0-1] | 1 [0-2] | 0.009 | 38 | 8 [5-10] | 8 [5-9] | 0.72 |

^a^Values expressed as median [Q1-Q3].

**^b^**P-values for comparing baseline with final HJHS from a linear mixed effects model.

**Supplementary Table S3.** Comparison of Baseline and Final Total HJHS

| Patients | Baseline Total HJHS | Final Total HJHS | P-value |
| --- | --- | --- | --- |
| N = 44 | 24 [16-38] ^a^ | 26 [15-35] **^b^** | 0.66 |

Total HJHS excludes global gait score

**^a^**Values expressed as median [Q1-Q3]

**^b^**P-values for comparing baseline with final total HJHS from Wilcoxon Signed Rank test

**^c^**Total HJHS excludes global gait score

**Supplementary Table S4.** Hemophilia Joint Health Score (HJHS) Characteristics by presence of hemarthrosis

| Joint  (N = 264) | Without hemarthrosis | | | With hemarthrosis | | |
| --- | --- | --- | --- | --- | --- | --- |
|  | **N** | **Baseline HJHS** | **Final HJHS** | **N** | **Baseline HJHS** | **Final HJHS** |
| Elbow | 84 | 2 [0-7] **^a^** | 2 [0-6] | 4 | 8 [6-9] | 10 [8-11] |
| Ankle | 80 | 6 [1-8] | 6 [3-9] | 8 | 6 [2-8] | 6 [4-8] |
| Knee | 84 | 2 [0-7] | 2 [1-7] | 4 | 4 [3-5] | 12 [6-15] |

**^a^**Values expressed as median [Q1-Q3]
